# From Encapsulation to Food Delivery: Application-Driven Design of Functional Ingredients Using Encapsulation and Coating Approaches for Future Food Systems

**DOI:** 10.3390/foods15183175

**Published:** 2026-09-08

**Authors:** Phatthranit Klinmalai, Pitiya Kamonpatana, Atcharawan Srisa, Phanwipa Wongphan, Khwanchat Promhuad, Anusorn Seubsai, Nathdanai Harnkarnsujarit

**Affiliations:** 1 Faculty of Agro-Industry, Chiang Mai University, Samut Sakhon 74000, Thailand; phatthranit.k@cmu.ac.th; 2 Department of Food Science and Technology, Faculty of Agro-Industry, Kasetsart University, Bangkok 10900, Thailand; fagipyk@ku.ac.th; 3 Department of Packaging and Materials Technology, Faculty of Agro-Industry, Kasetsart University, Bangkok 10900, Thailand; atcharawan.sri@ku.th (A.S.); phanwipa.w@ku.th (P.W.); khwanchatpromhuad@gmail.com (K.P.); 4 Department of Chemical Engineering, Faculty of Engineering, Kasetsart University, Bangkok 10900, Thailand; fengasn@ku.ac.th; 5 Center for Advanced Studies for Agriculture and Food (CASAF), Kasetsart University Institute for Advanced Studies (KUIAS), Kasetsart University, Bangkok 10900, Thailand

**Keywords:** encapsulation, food systems, food matrices, industrial processing, specialized food sectors

## Abstract

Encapsulation and coating approaches have become important tools in modern food systems for improving the stability, functionality, sensory quality, processability, and controlled delivery of bioactive and sensitive food ingredients. Their widespread adoption has enabled the incorporation of functional compounds into diverse food products while enhancing product quality, shelf life, and manufacturing performance. However, successful implementation depends not only on the encapsulation or coating strategy itself but also on the interactions among ingredient properties, carrier materials, food matrices, processing conditions, storage environments, and intended release behavior. Whereas recent reviews have mainly focused on specific encapsulation methods, carrier systems, industrial implementation, sensory functions, or regulatory aspects separately, this review integrates scientific publications and patent literature to examine method and system selection from food-engineering, formulation, processing, and industrial perspectives. Conventional processing and formulation approaches, including spray drying, freeze drying, coacervation, ionic gelation, emulsion-based encapsulation, and fluidized-bed coating, remain widely used, while established carrier systems such as liposomes and cyclodextrin inclusion complexes continue to support ingredient protection and delivery. Emerging carrier systems, including nanoemulsions, nanoliposomes, lipid nanoparticles, and hybrid multilayer structures, together with fabrication methods such as electrospraying and microfluidics, provide greater control over carrier architecture and release behavior but continue to face challenges related to manufacturing scalability, production throughput, storage stability, production cost, regulatory acceptance, and validation under industrial processing conditions. Although patent activity demonstrates continuing development of processing methods and carrier designs, patent publications alone do not establish commercial manufacture, market adoption, or industrial implementation. Across food applications, encapsulation improves ingredient protection, oxidation stability, sensory quality, dispersibility, controlled release, and process compatibility. By integrating research evidence with patent literature, this review further shows that recent progress is characterized primarily by application-driven refinement of carrier systems and fabrication methods rather than replacement of established approaches. Pet food is discussed as a representative specialized food application illustrating how encapsulation and coating strategies require adaptation to product format, processing severity, storage stability, palatability, and species-specific digestive requirements. Overall, this review highlights application-oriented food-engineering principles for selecting encapsulation methods and carrier systems suitable for industrial food applications.

## 1. Introduction

Encapsulation and coating approaches have become important strategies in modern food systems for improving the stability, functionality, and processability of sensitive food ingredients throughout manufacturing, storage, distribution, and gastrointestinal delivery. As functional ingredients, including probiotics, marine oils, vitamins, carotenoids, phenolic extracts, flavors, and essential oils, are increasingly incorporated into food products, maintaining their physicochemical stability and biological functionality has become a major challenge for food manufacturers. Many of these ingredients are highly susceptible to oxidation, thermal degradation, moisture, and interactions with surrounding food matrices, while some also exhibit poor dispersibility or undesirable sensory characteristics that limit their direct incorporation into food products [1,2,3,4,5]. Consequently, encapsulation together with post-processing coating has evolved beyond simple entrapment into a multifunctional food-engineering approach that enables oxidation control, thermal protection, flavor and odor masking, moisture management, dispersibility enhancement, and controlled release of bioactive ingredients [6,7,8,9].

To address these formulation and processing challenges, a wide range of encapsulation methods and carrier systems has been developed for food applications. Conventional processing and formulation methods, including spray drying, freeze drying, coacervation, emulsion-based encapsulation, ionic gelation, extrusion encapsulation, and fluidized-bed coating, remain widely used because they are compatible with diverse food matrices, scalable manufacturing processes, and existing food-production systems [10,11,12,13,14]. Established carrier systems, including liposomes and cyclodextrin inclusion complexes, are also widely used for ingredient protection and delivery. More recently, carrier systems such as nanoemulsions, nanoliposomes, solid lipid nanoparticles, nanostructured lipid carriers, and hybrid multilayer structures, together with fabrication methods such as electrospraying and microfluidics, have expanded opportunities for designing delivery systems with greater structural precision and tailored release characteristics. Nevertheless, their broader industrial implementation is still constrained by production cost, process scalability, long-term stability, regulatory acceptance, and product validation [15,16,17,18,19].

Importantly, the performance of an encapsulation system is determined not only by the selected fabrication or encapsulation method but also by the interactions among the encapsulated ingredient, carrier material, food matrix, processing conditions, water activity, lipid phase, storage environment, and intended release mechanism. Therefore, encapsulation strategies that perform successfully in one food product cannot be directly transferred to another without considering application-specific formulation and manufacturing requirements. This application-oriented perspective is increasingly important as encapsulation methods and carrier systems are adopted across a broader range of food products with distinct processing conditions, quality attributes, and functional requirements. Among these specialized food applications, pet food represents a valuable model for evaluating the translation of encapsulation approaches because it combines conventional food-processing principles with unique product formats and manufacturing constraints. Modern pet-food products include extruded dry kibble, wet foods, raw diets, toppers, powders, and chewable supplements, each requiring encapsulation systems capable of withstanding extrusion heat and shear, post-extrusion fat or flavor coating, prolonged ambient storage, variable moisture conditions, raw-diet safety concerns, and strong dependence on palatability [20,21,22,23]. Current research in this sector remains concentrated on a limited number of applications, particularly dry-kibble coating, probiotic snacks or supplements, and antimicrobial interventions for raw meat-based diets [20,21,24,25,26,27,28]. Rather than representing an independent technological field, pet food provides a representative example of how encapsulation systems developed for general food applications require adaptation to different product formats, processing conditions, storage environments, and species-specific performance requirements.

Although numerous review articles have summarized food encapsulation, most have focused on individual encapsulation methods, carrier materials, active compounds, industrial applications, sensory functions, or regulatory considerations separately. Only limited attention has been given to integrating these complementary perspectives to explain how encapsulation systems are translated from formulation design to practical food applications. In addition, relatively few reviews have considered scientific publications together with patent literature to evaluate both research development and industrial design directions. Therefore, this review critically evaluates conventional and emerging encapsulation approaches by integrating evidence from scientific publications and patents within a common food-engineering framework. Rather than considering conventional and emerging approaches as competing alternatives, the combined evidence indicates that recent progress is increasingly driven by application-specific carrier design, processing compatibility, and industrial feasibility. Pet food is included as a representative specialized food application to illustrate how general encapsulation principles are adapted to different processing conditions, product formats, storage environments, and functional requirements. Although other specialized food systems, such as clinical nutrition, infant nutrition, and foods for older adults, also present application-specific formulation challenges, pet food was selected because it integrates several engineering constraints within a single manufacturing sector. It is therefore used as an illustrative case study rather than as the primary subject of this review.

## 2. Literature and Patent Search Strategy

Among specialized food sectors, pet food was selected because it combines several formulation and processing challenges that are also encountered in human food systems, including thermal processing, post-processing coating, oxidative stability, sensory acceptance, storage stability, and functional ingredient delivery. Although the available literature is more limited than for conventional human foods, this application provides a practical case study for discussing how general encapsulation principles can be adapted to product-specific engineering constraints rather than representing a separate encapsulation technology. This review was conducted as a structured narrative review integrating evidence from scientific publications and patent literature to examine current directions in food encapsulation methods and carrier systems and their applications in food systems. Scientific publications were used to evaluate reported formulation performance, whereas patent documents were reviewed to identify claimed carrier designs, processing strategies, product concepts, and industrial directions. The purpose of the review was to provide an application-oriented synthesis of the available evidence rather than a quantitative systematic review or meta-analysis.

Scientific publications were primarily retrieved from the Scopus database. Searches were conducted using combinations of keywords related to encapsulation methods and carrier systems (e.g., encapsulation, microencapsulation, nanoencapsulation, spray drying, freeze drying, coacervation, ionic gelation, liposome, cyclodextrin, nanoemulsion, nanoliposome, solid lipid nanoparticle, nanostructured lipid carrier, electrospray, electrohydrodynamic processing, microfluidics, and hybrid systems) together with food-related terms (e.g., food, beverage, dairy, bakery, nutraceutical, confectionery, snack, and functional food). Additional searches were performed according to the objectives of individual tables, including searches focused on method and carrier-system classification, active-compound categories, and specialized pet-food applications. Search results were initially sorted using the database relevance ranking and only English-language research articles were considered. Publications were screened by title and abstract, followed by full-text evaluation when required to determine their relevance to the scope of this review. Studies were included only when the encapsulation system was directly relevant to food applications and provided sufficient technical information for qualitative comparison.

Patent information was retrieved from the WIPO PATENTSCOPE database using a comparable keyword strategy to facilitate parallel evaluation of scientific publications and patent literature, which indexes international (PCT) applications together with participating national and regional patent collections. Searches combined food-related terms with encapsulation-related keywords and application-specific keywords according to the objective of each table, including method and carrier-system classification, active-compound categories, formulation functions, and specialized pet-food applications. Patent documents were screened by title, abstract, claims, and technical description to assess their relevance to food encapsulation. When multiple records belonged to the same patent family, a representative document was selected to avoid duplication. Equivalent filings or continuations within the same patent family were represented by a single document whenever applicable to avoid repeated interpretation of the same invention. Patent documents were interpreted as sources of claimed technical solutions, carrier designs, processing concepts, and industrial directions rather than as direct evidence of experimental performance. Patent legal status (e.g., granted, pending, expired, or abandoned), technology-readiness level, licensing status, commercial manufacture, production capacity, economic feasibility, and market adoption were not used as selection criteria because these data are not consistently reported across patent documents or corresponding scientific literature. Accordingly, patent evidence in this review is interpreted qualitatively to illustrate technological development rather than commercialization status. The searches were conducted up to June 2026 and covered publications and patent documents published between 2016 and 2026. Studies and patent documents were included when they described food-grade encapsulation systems, carrier materials, encapsulation methods, formulation strategies, food applications, or specialized food sectors relevant to the scope of this review. Publications focusing exclusively on pharmaceutical, biomedical, cosmetic, agricultural, or non-food industrial applications without clear relevance to food systems were excluded. Conference abstracts, editorials, duplicate records, and documents lacking sufficient technical information were also excluded after screening. The selected evidence was synthesized qualitatively to compare established and emerging encapsulation methods and carrier systems, formulation strategies, reported functional performance, and industrial design trends across food systems.

## 3. Encapsulation and Coating Methods and Carrier Systems

Encapsulation is a formulation approach used to improve the stability, compatibility, and functional performance of sensitive active compounds in food systems. Because encapsulation platforms differ in structure, wall materials, scalability, release behavior, and process tolerance, method and carrier-system selection must consider the active compound, carrier system, food matrix, processing condition, storage requirement, and intended delivery function [7,9].

### 3.1. Classification of Food Encapsulation Methods and Carrier Systems

Food encapsulation and coating approaches include diverse fabrication methods, carrier structures, and delivery systems that differ in structural organization, particle scale, release behavior, and industrial implementation. Consequently, no single classification can adequately represent all encapsulation approaches because they may be categorized according to fabrication method (e.g., spray drying or freeze drying), carrier architecture (e.g., liposomes, hydrogel beads, and multilayer structures), particle size (micro- or nanoscale systems), release mechanism, or intended application. These classification schemes frequently overlap, and a single carrier system may belong to multiple categories depending on its formulation design and processing conditions. In the present review, encapsulation methods and carrier systems are organized using a practical application-based framework based on their current use in food systems. This approach facilitates comparison of formulation strategies, carrier materials, industrial feasibility, and food applications. Within this framework, established fabrication and processing methods include spray drying, freeze drying, coacervation, ionic gelation, extrusion encapsulation, emulsion-based encapsulation, and fluidized-bed coating. Established carrier systems include liposomes and cyclodextrin inclusion complexes. These methods and systems remain widely applied because they are compatible with food-grade materials, existing manufacturing infrastructure, and diverse food matrices. Continued development has focused primarily on improved carrier design, wall-material selection, process optimization, and application-specific formulation. Emerging carrier systems include nanoemulsions, nanoliposomes, solid lipid nanoparticles, nanostructured lipid carriers, and hybrid multilayer structures, whereas fabrication methods include electrospraying, electrohydrodynamic processing, microfluidics, and supercritical-fluid processing [6,7,8,9]. These approaches provide greater flexibility for tailoring particle architecture, interfacial properties, loading efficiency, and release behavior. Their wider application continues to depend on manufacturing scalability, production throughput, storage stability, regulatory acceptance, economic feasibility, and validation under industrial processing conditions. Because technology-readiness level, commercial manufacture, licensing status, production cost, energy consumption, and market adoption are only rarely reported consistently across research publications and patent documents, these aspects could not be systematically compared and therefore should not be inferred from publication activity alone.

### 3.2. Research Evidence on Food Encapsulation Methods and Carrier Systems

As summarized in Table 1, established encapsulation methods and carrier systems remain important because they offer different balances between industrial feasibility, ingredient protection, and matrix compatibility. Spray drying is widely used for powders, premixes, and coating ingredients because it is scalable, cost-effective, and compatible with carbohydrate- and protein-based wall materials. However, elevated drying temperatures can limit its use for highly heat-sensitive actives [1,11]. Freeze drying better preserves thermolabile compounds, especially probiotics and high-value bioactives, but high cost and long processing time restrict large-scale use [11,29].

Other conventional systems are selected according to the target function. Coacervation and ionic gelation form beads, shells, or hydrogel matrices that are useful for probiotic protection and controlled release, although pH, ionic strength, and mechanical stress can affect performance [5,30,31,32]. Emulsion-based systems are more suitable for lipophilic compounds because they improve dispersibility and compatibility with wet or lipid-containing matrices, but coalescence and phase separation remain common limitations [33,34]. Fluidized-bed coating, liposomes, and cyclodextrin inclusion complexes also support post-processing delivery, flavor masking, solubility improvement, and delivery of hydrophilic or lipophilic compounds, although cost, stability, and loading capacity may limit broader application [4,14,35,36,37,38].

Emerging carrier systems and fabrication methods extend these functions through smaller particle size, more controlled structures, and tunable release behavior. Nanoemulsions and nanoliposomes are mainly used to improve dispersibility and delivery of hydrophobic bioactives, while solid lipid nanoparticles and nanostructured lipid carriers can protect lipid-based compounds and support-controlled release [4,37]. Electrospraying, electrohydrodynamic processing, and microfluidics provide better control of capsule morphology and particle size, but industrial use remains limited by low throughput, equipment cost, and scale-up challenges [17]. Hybrid and multilayer systems combine polymeric, lipid, and biopolymer layers to provide multiple functions, such as protection, flavor masking, and delayed release, although their value still depends on processing compatibility and validation in the target food format [33,36]. Overall, established and emerging encapsulation approaches provide different functional and manufacturing advantages. Their main characteristics are summarized in Table 1, while their application-oriented selection is consolidated in the framework presented in Section 7.

### 3.3. Patent Evidence on Food Encapsulation Methods and Carrier Systems

Complete patent records describing established and emerging encapsulation methods, coating processes, and carrier systems are provided in Supplementary Tables S1 and S2. The main-text discussion therefore focuses on representative design directions rather than reproducing the complete patent inventory. Unlike research publications, patent documents primarily disclose claimed technical concepts, formulation strategies, processing routes, and intended functions rather than independently validated formulation performance. Accordingly, patent evidence is interpreted here as evidence of formulation and process development rather than confirmation of commercial manufacture or industrial implementation.

Among established approaches, spray-drying patents use protein-, starch-, or polysaccharide-based matrices to improve dispersibility, formulation compatibility, and active retention, whereas some extract-based systems primarily use spray drying for stabilization and powder production [10,12,13]. Freeze-drying patents remain concentrated on heat-sensitive biological ingredients, including probiotics, peptides, and fungal-derived materials [39,40,41]. Coating-related patents describe fluidized-bed coating, curtain coating, hard-panning, dry-particle coating, starch–fat coating, and solid-fat shell systems for surface delivery, barrier formation, moisture control, volatile retention, or structural stabilization [42,43,44,45,46,47]. Emulsion-based, lipid-matrix, and inclusion systems provide related approaches for volatile retention, particle distribution, protection of moisture-sensitive actives, solubility improvement, and bitterness masking [48,49,50,51].

Emerging patent evidence includes nanoemulsions, protein-based nanocomplexes, polymer-coated nanoparticles, peptide condensates, pigment-stabilization systems, and electrospun nanofibers [15,18,19,52,53,54,55]. These examples indicate increasing control over carrier architecture and release behavior, but their practical application remains dependent on product-specific validation, manufacturing feasibility, and regulatory acceptance. Representative patent-derived design trends from these and application-specific patents are summarized in Table 2, while the complete patent records are provided in Supplementary Tables S1–S3.

## 4. Applications of Encapsulation and Coating Approaches in Food Systems: Research Publications and Patents

Food-application evidence was evaluated by active-compound group because each group presents different formulation challenges and delivery requirements. Across the reviewed studies, encapsulation was mainly applied to improve protection of sensitive ingredients, matrix compatibility, sensory quality, dispersibility, and release behavior. Direct comparison remains limited because carrier materials, food matrices, processing conditions, storage protocols, and performance measurements differed considerably across studies [1,4,5,33,34,56,57,58,59,60]. Representative research evidence is therefore summarized in Table 3, while the following discussion focuses on broader formulation and application trends.

### 4.1. Research Evidence on Encapsulation Applications in Food Systems

Representative research evidence for probiotics, marine oils, vitamins, carotenoids, phenolic or botanical compounds, and flavors or essential oils is summarized in Table 3. The table retains selected studies that illustrate different carrier systems, food matrices, and measurable outcomes, while additional studies remain incorporated in the narrative synthesis. Probiotics and live microbial cultures are among the most established applications. Their main limitation is loss of viability during drying, heating, storage, reconstitution, and gastrointestinal passage. Multilayer alginate–whey protein systems, Pickering emulsions, spray drying, extrusion-based beads, hydrogel microcapsules, and synbiotic matrices have improved survival of Lactobacillus and Bifidobacterium strains under gastric, thermal, storage, or food-processing conditions [11,29,30,32,33,61,62]. These studies suggest that probiotic performance depends not only on barrier formation but also on the interaction between the wall material, encapsulation process, and target food matrix. Reported survival varied with probiotic strain, carrier composition, drying conditions, and storage environment, indicating that no single encapsulation strategy was consistently superior across all applications.

Marine oils and omega-3 lipid ingredients require protection against oxidation, rancidity, fishy odor, poor aqueous dispersibility, and loss of EPA and DHA. Nanoliposomes, spray-dried emulsions, chitosan nanoparticles, and phospholipid carriers have improved oxidative stability, odor masking, encapsulation efficiency, and sensory acceptance of fish oil, shrimp oil, and krill oil in fortified foods [1,4,37,63,64]. However, the reported improvements depended on carrier composition, drying or emulsification conditions, storage conditions, and food matrix, which limits direct comparison among formulations.

Vitamins, lipid-soluble micronutrients, carotenoids, and natural pigments are mainly limited by poor water solubility and degradation under oxygen, light, heat, and pH changes. Gum-arabic encapsulation improved vitamin D3 stability, bioaccessibility, and absorption in beverage systems, while protein–polysaccharide nanoparticles, complex coacervation, ionic gelation, spray drying, and electrospinning improved dispersibility, color retention, thermal stability, water solubility, antioxidant activity, or bioaccessibility of carotenoid and pigment systems [3,5,17,65,66,67,68]. Different studies evaluated different performance endpoints, including retention, antioxidant activity, color stability, and bioaccessibility, which limits direct quantitative comparison.

Phenolic extracts and botanical bioactives often show poor solubility, bitterness, astringency, oxidation, matrix interaction, and limited bioaccessibility. Liposomes, nanoliposomes, nanoemulsions, niosomes, microemulsions, and protein–cyclodextrin nanocomplexes have improved stability, taste masking, solubility, oxidative protection, or intestinal release in food matrices [36,38,69,70,71,72]. Because the investigated compounds, carrier systems, and food applications differed substantially, the reported outcomes should be interpreted within the context of each formulation.

Flavors, aroma compounds, and essential oils are commonly encapsulated because they are volatile, pungent, poorly soluble, or sensitive to processing and storage. Cyclodextrin inclusion complexes, spray-dried powders, ionic-gelation nanoparticles, freeze-dried nanocapsules, protein–polysaccharide nanocomplexes, and Pickering emulsions have improved retention, odor masking, thermal stability, water dispersibility, controlled release, antimicrobial activity, or aroma release [2,14,34,35,73,74,75,76]. Aroma retention and sensory performance nevertheless remained dependent on carrier composition, processing conditions, and food application.

**Figure 1:**
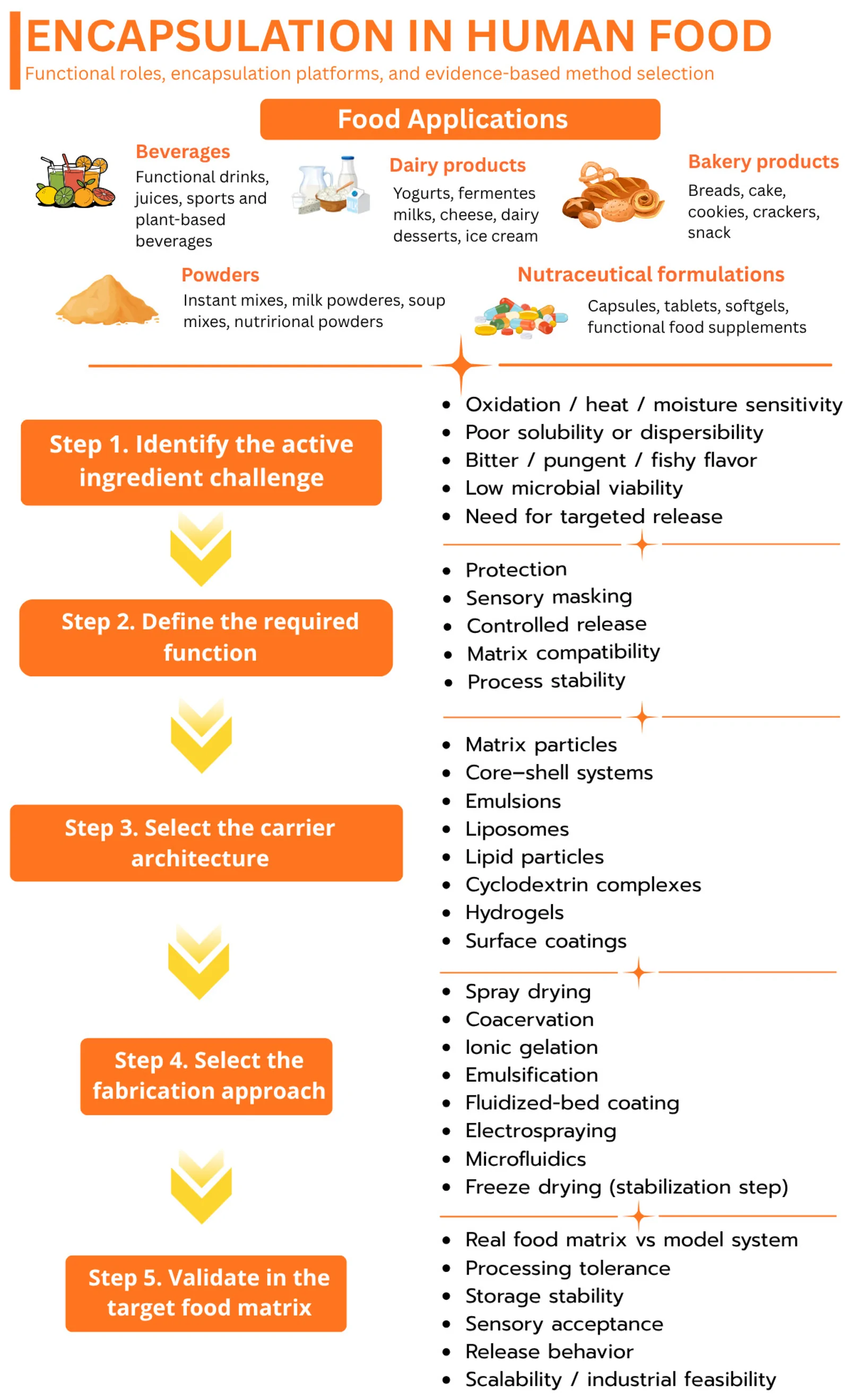
Application-oriented decision framework for selecting encapsulation and coating approaches in food systems. Graphical elements were sourced from Canva Pro and used under the Canva Content License Agreement.

Overall, the representative studies summarized in Table 2 show that encapsulation can provide protection, sensory modification, improved dispersibility, and controlled release across different active-compound groups. However, differences in formulations, food matrices, processing conditions, and evaluation methods limit direct comparison among studies. The functional roles identified from the broader evidence base are summarized in Figure 1, while their application-oriented selection is consolidated in Section 7.

### 4.2. Patent Landscape of Encapsulation Methods and Carrier Systems in Food Applications

Unlike the research evidence summarized in Section 4.1, the patent literature reviewed in this section reflects claimed technical solutions rather than independently validated formulation performance. Patent evidence is therefore interpreted as complementary information describing formulation and process-development directions rather than confirmation of commercialization or industrial implementation. Across the reviewed records, four recurring formulation objectives were identified: protection of unstable lipids and bioactive compounds, improved dispersibility of hydrophobic ingredients, masking of undesirable taste or odor, and controlled release in food or supplement formats. Representative examples of these patent-derived design trends are summarized in Table 3, while the complete application-based patent inventory is provided in Supplementary Table S3.

**Table 2:** Representative patent-derived design trends in food encapsulation and coating.

Design Objective	Representative Method/Carrier	Representative Patent	Claimed Function	Evidence Interpretation	Ref.
Oxidation protection	Spray drying/complex coacervation; protein–polysaccharide wall	WO2017197453	Improved oxidative stability of omega-3 PUFA	Patent-claimed function	[56]
Dispersibility	Spray-dried flavonoid dispersion; phosphate-assisted/protein carrier	WO2022162565	Improved aqueous solubility and dispersion of hydrophobic flavonoids	Patent-claimed function	[59]
Sensory masking/delayed release	Multilayer core–shell particles; alginate/cellulose-based carriers	WO2022013548	Reduced taste impact; heat protection; delayed gastric release	Patent-claimed function	[58]
Modified release	Hot-melt-extruded polymeric matrix	US20200179477	Taste masking; sustained release of cacao flavonoids/catechins	Patent-claimed function	[77]
Probiotic protection	Multilayer coated probiotic particles	EP4042881	Improved storage stability and resistance to gastric acid/moisture	Patent-claimed function	[78]
Flavor retention/sustained release	Seed-based carrier	US20210186073	Improved oxidative stability, flavor retention, and delayed release	Patent-claimed function	[79]

**Table 3:** Representative research evidence on encapsulation methods and carrier systems in food applications.

Active/Group	Method/Carrier	Food Matrix or Test System	Quantitative Outcome/Study Condition	Ref.
*Lactobacillus rhamnosus* GG	Extrusion; sodium alginate-based matrices	Reconstituted product	Encapsulation efficiency of 89.85–92.28%; cells maintained above 7 log CFU g^−1^ after 45 days at 4 and 25 °C	[29]
*Lactobacillus plantarum*	Extrusion-based hydrogel beads; alginate–gelatin	Simulated gastrointestinal conditions	Encapsulation efficiency of 97.7%; improved gastric protection and intestinal release	[32]
Shrimp oil rich in EPA, DHA, and astaxanthin	Nanoliposome; soy lecithin	Storage study	Improved oxidative stability during 8-week storage; EPA, DHA, and astaxanthin retained	[37]
Fish oil rich in EPA and DHA	Nanoliposome; lecithin phospholipid bilayer	Bread fortification	Encapsulation efficiency of about 90.12%; improved storage stability; no undesirable odor or taste reported	[64]
Vitamin D3	Gum arabic encapsulation followed by freeze drying	Beverage	Stable at pH 2.0–7.4 during 100-day refrigerated storage; bioaccessibility 95.76%; 48 h AUC was 4.32-fold higher than nonencapsulated vitamin D3	[3]
Lutein	Antisolvent nanoparticles; zein–soluble soybean polysaccharide	Model/functional system	Encapsulation efficiency above 80%; solubility increased more than 30-fold; retention 96.27% after 15 days	[68]
β-carotene and palm-oil carotenoids	Complex coacervation/ionic gelation; chitosan-based carriers	Yogurt and bread	Encapsulation efficiency above 95%; carrier affected simulated digestive release	[5]
Quercetin	Nano-niosome; Span 60/Tween 80 with phytosterol or PEG 400	Orange juice	Vesicle size of 86–149 nm; encapsulation efficiency up to 95.68%; evaluated during 30 d storage and after pasteurization	[71]
EGCG	Self-assembled nanocomplex; whey protein isolate–β-cyclodextrin	Simulated gastrointestinal conditions	Encapsulation efficiency of 79.61%; reduced gastric release and increased intestinal release	[70]
Cumin essential oil (CSO)	Ionic gelation; chitosan–tripolyphosphate nanoparticles	Mayonnaise	Particle size 269–326 nm; reduced peroxide value and thiobarbituric acid value during storage	[73]

For lipid- and algae-derived ingredients, patents combine established encapsulation methods with modified carrier architectures intended to improve oxidative stability, dispersibility, sensory quality, and compatibility with different food matrices [16,56,60,80,81,82,83]. These examples indicate a shift from ingredient protection alone toward formulations that combine stabilization with matrix compatibility, sensory modification, and product-specific delivery. However, these functions should be interpreted as patent-derived design objectives rather than evidence of commercial manufacture or industrial implementation.

Patents involving cannabinoids, phenolics, flavonoids, and mixed plant bioactives mainly address poor aqueous solubility, instability, sedimentation, bitterness, and limited compatibility with beverages or fortified foods. Proposed systems include liposomes, spray-dried powders, hot-melt-extruded matrices, protein-based carriers, multilayer particles, casein-based nanocarriers, mycelium-based matrices, and plant-derived multilayer structures [57,58,59,77,84,85,86,87]. These records indicate interest in multifunctional carrier systems, although the claimed functions remain dependent on carrier composition, active-compound properties, and intended food application.

For probiotics, patent documents describe multilayer coatings, plant protein–carbohydrate matrices, spontaneous alginate hydrogels, and lipid microcapsules intended to improve survival during processing, storage, and gastrointestinal exposure [78,88,89,90]. Patent records related to flavors and essential oils similarly describe seed-based carriers, hydrogel microcapsules, β-cyclodextrin inclusion systems, and plant protein–polysaccharide coacervates for volatility control, oxidation protection, taste masking, and controlled release [79,91,92,93,94]. These patent-derived concepts complement the research literature by illustrating formulation-development directions but should not be interpreted as independently verified performance. Manufacturing scale, production throughput, economic feasibility, regulatory approval, licensing status, and market adoption also remain insufficiently reported for systematic comparison.

## 5. Design Principles for Translating Food Encapsulation and Coating Approaches to Specialized Food Systems

Encapsulation approaches developed for food applications can be adapted to specialized food sectors when the same formulation functions are required under different processing, storage, or consumption conditions. The key issue is not direct transfer from one product category to another, but design adaptation based on the active compound, carrier system, matrix composition, processing route, storage environment, sensory requirement, and release target. This section interprets the reviewed evidence through five design functions: protection, flavor or odor masking, controlled release, dispersibility, and process compatibility.

### 5.1. Protection of Sensitive Actives

Food studies show that encapsulation can protect probiotics, marine oils, vitamins, carotenoids, and other bioactives from heat, oxidation, moisture, and gastrointestinal stress. For probiotics, alginate–whey protein systems, Pickering emulsions, spray-dried powders, extrusion-based beads, and hydrogel microcapsules have improved survival during processing, storage, and simulated digestion [11,29,33,61]. Similar protective roles have been reported for marine oils and vitamin D3, where encapsulation improved oxidative stability, odor control, and delivery performance [3,4,37].

For specialized food sectors, this function is most relevant when active ingredients are exposed to thermal treatment, drying, coating, long storage, or digestion. Pet food provides a useful example because sensitive ingredients may encounter extrusion, drying, post-processing fat application, and extended ambient storage. Early studies support protection in probiotic snack coatings, probiotic supplement powders, and encapsulated organic acids for raw meat-based diets [24,27,28]. However, protection shown in beverages or supplement powders cannot be assumed to represent survival in all food formats. More realistic adaptation routes include post-processing coating, protected premixes, soft matrices, wet-food inclusions, and freeze-dried formats.

### 5.2. Flavor or Odor Masking

Food research shows that encapsulation can reduce pungent, bitter, fishy, or otherwise undesirable sensory properties. Cyclodextrin-based microcapsules have improved odor control and release behavior of garlic essential oil, while nanoliposomal fish-oil systems improved oxidative stability and reduced fishy perception in fortified foods [4,35]. Pickering emulsions have also been used to reduce aroma loss and regulate flavor release in plant-based meat analogs [34].

This function is important in specialized food systems where functional ingredients may affect acceptance. Encapsulated oils, botanical extracts, vitamins, minerals, hydrolyzed proteins, probiotics, and palatability compounds can change aroma, taste, or aftertaste. Patent evidence indicates that cyclodextrin inclusion, coacervation, hydrogels, lipid matrices, seed-based carriers, and coating methods are used to manage bitterness, astringency, fishy odor, volatile loss, and off-flavor development [49,58,79,87,91,93,94]. Cyclodextrins could support taste modulation, whereas coacervates, lipid matrices, hydrogels, and seed-based carriers provide stronger physical retention and delayed flavor release. Application still requires sensory and storage validation in the target product format.

### 5.3. Controlled or Targeted Release

Controlled release is important when encapsulation is designed to reduce premature release and support delivery at a later processing, storage, or digestive stage. In food studies, probiotic beads, multilayer hydrogels, and related gel systems have reduced gastric release and improved intestinal release for Lactobacillus systems, garlic essential oil, and chia phenolic extract [29,32,35,36].

In specialized food sectors, controlled release is most relevant to probiotics, postbiotics, enzymes, botanicals, and gut-health additives. Pet food again provides a useful case because release behavior may be affected by product format and species-specific digestion. Probiotic coatings and microcapsules in dried dog snacks and supplement powders improved gastric protection and simulated gastrointestinal survival [24,28]. Patent evidence also supports this function through gastro-resistant coatings, multilayer probiotic particles, lipid microparticles, and alginate hydrogels designed to delay release or protect actives before intestinal delivery [43,58,78,87,89,90]. Translation still requires digestion models and product-format validation that match the intended application.

### 5.4. Dispersibility and Matrix Compatibility

Encapsulation improves dispersibility and matrix compatibility of hydrophobic or unstable compounds in food systems. Gum-arabic encapsulation improved vitamin D3 dispersion and stability in beverages, while zein–soluble soybean polysaccharide nanoparticles improved lutein dispersibility, colloidal stability, and bioaccessibility [3,68]. Coacervate, ionic-gel, and liposome-based systems have also supported the incorporation of carotenoids and phenolic extracts into yogurt, bread, muffins, and related food matrices [5,36,67].

This function translates to food formats that require uniform distribution of active ingredients, including beverages, emulsions, gravies, powders, premixes, coatings, and supplements. Patent evidence further shows that emulsion systems, cyclodextrins, nano-complexes, and protein–polysaccharide carriers can improve distribution of omega-3 oils, flavonoids, algae-derived compounds, proteins, and flavor ingredients [18,59,60,80]. In specialized food products, dispersibility also affects dose uniformity, coating quality, texture, and sensory consistency. Direct evidence remains product-specific, so matrix validation is still needed before broader application.

### 5.5. Process Compatibility

Process compatibility is one of the most practical design principles in food encapsulation. In food studies, spray-dried probiotics, multilayer gel particles, lipid carriers, and flavor or oil systems have improved survival or retention during drying, heating, baking, pasteurization, storage, or refrigerated holding [4,11,29,34].

In specialized food sectors, process compatibility is best evaluated by product format and point of addition, not only by active type. In pet food, for example, post-processing coating is often more realistic for heat-sensitive probiotics and flavors, whereas lipid nutrients, botanicals, and other functional ingredients may be incorporated through protected premixes, wet-food systems, soft-chew matrices, or supplement powders. Current studies support post-processing applications such as organic-acid coatings, β-hydroxy-β-methylbutyrate coatings, menhaden fish-oil coatings, and medium-chain fatty acid treatments for microbial control on dry kibble [20,21,25,26]. Probiotic coatings and microencapsulated probiotic powders also improved survival during drying, freeze-drying, chilled storage, and simulated gastric exposure in dog-oriented formulations [24,28]. This framing keeps translation practical without assuming that performance in one food matrix will be repeated under different processing conditions.

## 6. Pet Food as a Representative Specialized Food Application

Pet food provides a useful representative case for examining how food encapsulation principles are adapted to specialized product formats. Compared with general food applications, direct research on encapsulation and coating approaches in pet food remains limited and concentrated in a few practical areas. Representative research evidence is summarized in Table 4 and mainly addresses post-processing microbial control in dry kibble, probiotic stabilization in snacks or supplement powders, and safety interventions in raw meat-based diets [20,21,24,25,26,27,28]. Patent evidence covers a broader range of dry, wet, liquid, powder, capsule, and chewable formats; a patent-derived summary of these application areas is provided in Supplementary Table S4.

### 6.1. Dry Pet Food, Kibble, and Extruded Products

Dry pet food is the most developed application area because kibble provides a clear need for post-extrusion delivery. Ingredients exposed to extrusion, drying, and long storage may lose activity or sensory quality; therefore, heat-sensitive components are often more practically applied through surface coating after the main thermal process. Current research mainly supports this use in microbial control. Organic-acid mixtures, β-hydroxy-β-methylbutyrate, menhaden fish oil, and medium-chain fatty acids have been applied to coated kibble to reduce Salmonella, STEC, or related microbial risks during storage [20,21,25,26]. These examples show that coating can serve both palatability and safety functions, although acidic or fatty-acid coatings may require palatability adjustment.

Patent evidence extends this concept from antimicrobial coating to broader surface and carrier design. Hydrocolloid glazes, fat-containing palatant coatings, low-temperature lipid multilayers, freeze-dried surface inclusions, pork-skin emulsions, and vacuum-assisted lipid coatings have been proposed to improve coating uniformity, aroma retention, palatability, moisture control, or delivery of probiotics and fat-soluble nutrients [22,23,95,96,97,98,99,100]. Some patents also describe in-process protection of vitamins or probiotics using oligopeptide-wall microcapsules or reinforced alginate–gelatin systems [101,102]. However, post-extrusion coating remains the more practical strategy for heat-sensitive actives because it minimizes thermal exposure while remaining compatible with existing industrial manufacturing. Selection of coating materials should additionally consider feed-grade ingredient approval, oxidative stability during storage, palatability, and manufacturing consistency because carrier systems acceptable for human foods may require separate authorization or product-specific evaluation in companion-animal feeds depending on the target jurisdiction.

### 6.2. Wet Pet Food and Liquid Supplements

Wet pet foods and liquid supplements represent a different application challenge because the matrix is high in moisture and often requires texture stability, phase stability, sterilization tolerance, and taste masking. Direct research evidence remains limited. The strongest example is the use of encapsulated citric and lactic acids in raw meat-based dog diets, where encapsulated acidulants reduced *Salmonella* while preserving product quality better than dry-plated acids at similar inclusion levels [27]. Although raw diets differ from conventional canned foods, this study illustrates a broader formulation principle: encapsulation can moderate the interaction between antimicrobial activity and product quality in high-moisture systems.

Patent evidence suggests wider possible functions in wet and liquid formats. Encapsulated or gelled systems have been proposed to improve dispersion of lipophilic actives, reduce bitterness or off-flavor, stabilize color and texture, and protect probiotics or oral actives during storage and digestion. Examples include calcium-crosslinked beta-carotene emulsion gel in wet canned cat food, beta-cyclodextrin–whey protein systems for hemp extract incorporation, water-in-oil-in-water probiotic emulsions, coated particles for bitterness masking, and cyclodextrin-assisted solubilization in companion-animal liquid formulations [103,104,105,106]. These examples align with broader food evidence on emulsions, inclusion complexes, and hydrogel systems, but direct food-matrix validation in pet products remains limited. Although these formulation concepts are consistent with broader food encapsulation research, practical application in commercial wet pet foods requires validation after sterilization or retort processing together with assessment of ingredient compatibility, feed-additive authorization where applicable, storage stability, and species-specific safety.

### 6.3. Powder, Capsule, Snack, and Chewable Formats

Powder, capsule, snack, and chewable formats allow encapsulation to be applied under less severe processing conditions than conventional kibble. Current research is strongest for probiotic protection. In dried dog snacks, sodium-alginate hydrogel coatings improved probiotic survival during chilled storage and simulated gastrointestinal digestion, while alginate-based microencapsulation in dog supplement powders improved survival after freeze drying, storage, and simulated gastric exposure [24,28]. Representative pet-food research evidence is summarized in Table 4.

Patent documents additionally describe hydrocolloid-coated palatant powders, sustained-release microcapsules, probiotic sachets, high-internal-phase fish-oil emulsions, dual-layer coated nutritional supplements, linseed-oil microcapsules, and freeze-dried probiotic microcapsules for powders, capsules, soft chews, and semi-moist products [107,108,109,110,111,112,113]. A patent-derived summary of pet-food application formats is provided in Supplementary Table S4. These patent records describe proposed formulation routes and should not be interpreted as evidence of commercial implementation.

### 6.4. Comparative Interpretation of Application Limits, Regulatory Requirements, and Design Needs

Across research articles and patents, encapsulation addresses similar formulation problems in general food systems and specialized sectors: loss of activity, oxidation, poor dispersibility, undesirable taste or odor, and uncontrolled release during storage or digestion [1,4,7,9]. The main difference is the design target. General food systems often begin with the active ingredient and the eating matrix, such as beverages, dairy products, bakery systems, powders, or supplements. Pet-food systems more often begin with the product format and manufacturing route. Dry kibble is porous, heated, dried, fat-coated, and stored for long periods; wet foods require stable dispersion and texture during sterilization or storage; powders and chews require dose control, texture stability, and voluntary intake.

For this reason, application to pet food is best guided by point of addition and desired function. Surface coating fits kibble finishing, microbial control, and palatant delivery. Emulsion and double-emulsion systems fit wet foods and liquid supplements. Dry matrices, capsules, and soft-chew microcapsules fit dose-oriented supplement formats. However, good performance in one food matrix does not ensure the same result in another because extrusion, low water activity, fat coating, long storage, and animal acceptance create different constraints. Controlled-release data also need species-appropriate validation before being applied to dogs or cats [24,28,43,78,89,90].

Regulatory assessment should consider the complete encapsulated system rather than the active ingredient alone. The active compound, wall material, carrier, emulsifier, coating material, crosslinking agent, and other formulation components must be suitable for the intended food or feed use and for the target market. Depending on the jurisdiction and intended use, an ingredient or carrier may need to meet applicable food-additive requirements or be evaluated under frameworks such as GRAS or novel-food authorization. Nano-enabled systems require additional attention because definitions and labeling requirements for engineered nanomaterials are not uniform among jurisdictions. Processing methods using organic solvents also require control of residual solvent levels in the final product. In addition, safety evaluation should consider whether the carrier remains intact, migrates from the encapsulated structure into the food matrix, or is degraded or released during processing and gastrointestinal digestion. These factors are relevant to exposure assessment and dose control, particularly for systems designed to increase bioaccessibility or provide sustained release. Regulatory acceptance in one jurisdiction should therefore not be assumed to establish acceptance in another, and the regulatory status of the complete formulation should be confirmed for the intended product and market. Pet-food applications require additional evaluation because suitability for human food does not by itself establish suitability for companion-animal feeding. Carrier materials and functional additives should be evaluated for species-specific safety and, where applicable, feed-additive authorization. Product development must also consider palatability and voluntary intake because an encapsulated ingredient that is technically stable but reduces food acceptance may not provide the intended dose. Dose-oriented powders, capsules, supplements, and chews additionally require control of dose uniformity and stability throughout shelf life. The intended function and claims also require attention because the regulatory boundary between a nutritional or feed product and a veterinary therapeutic product differs among jurisdictions. These considerations are particularly important when encapsulation is used for sustained release, gastrointestinal targeting, antimicrobial functions, or other applications that may extend beyond conventional nutritional delivery.

**Figure 2:**
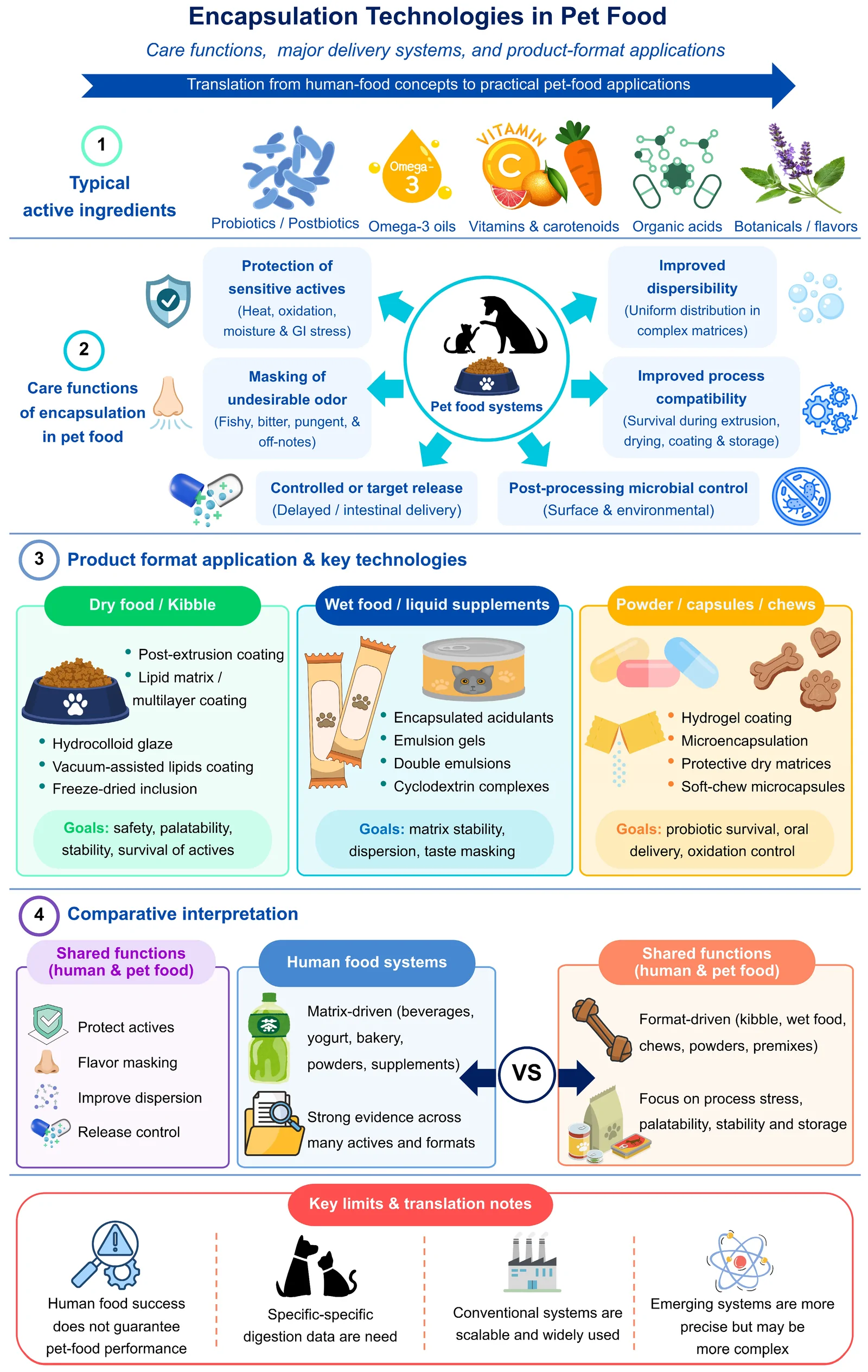
Application-oriented framework for translating encapsulation and coating approaches to pet-food product formats. Graphical elements were sourced from Canva Pro and used under the Canva Content License Agreement.

The pet-food evidence shows that encapsulation strategies must be adapted to product format, point of addition, species-specific safety, palatability, and intended use. Conventional and emerging systems may both be applicable, but patent activity alone does not demonstrate regulatory approval, commercial implementation, or industrial adoption. The main product-format applications and translation limits are summarized in Figure 2, while the general method- and carrier-selection framework is presented in Section 7.

## 7. Future Perspectives and Application-Oriented Selection Framework

The reviewed evidence indicates that future development should focus on application-oriented selection rather than increasing carrier complexity alone. A practical selection framework can be organized as a sequence of linked considerations. First, the required function should be defined from the limitation of the active ingredient, such as protection from oxidation or heat, sensory masking, improved dispersibility, or controlled release [7,9]. Second, the carrier and encapsulation method should be compatible with the physicochemical properties of the active compound and the target food matrix. Third, the formulation should be evaluated under the actual manufacturing route and point of addition because extrusion, drying, heating, homogenization, coating, and other operations impose different stresses. Fourth, performance should be validated during the intended storage period and, where relevant, under appropriate digestion or release conditions. Finally, practical implementation requires acceptable sensory quality, regulatory suitability, manufacturing scalability, and economic feasibility for the intended product.

Within this framework, established encapsulation methods and carrier systems remain appropriate when manufacturing compatibility, throughput, and practical implementation are major requirements. Spray drying, freeze drying, ionic gelation, and coating represent established processing or fabrication methods, whereas emulsion and lipid-based systems represent carrier or delivery formats. Emerging carrier systems, including nanoemulsions, nanoliposomes, lipid nanoparticles, and multilayer structures, together with fabrication methods such as electrohydrodynamic processing, microfluidics, and supercritical-fluid processing, may provide greater control over particle architecture and release behavior. However, this additional structural control requires product-specific validation before broader food application.

The reviewed scientific and patent literature also indicates increasing interest in multifunctional carriers that combine protection, sensory modification, dispersibility, and release control [58,80,81,86,92]. However, patent disclosures describe claimed technical solutions and should not be interpreted as evidence of industrial manufacture or commercial adoption. Further work is therefore needed to validate promising systems under realistic processing and storage conditions and, where appropriate, at industrial scale. For specialized food applications, including pet food, the same framework can be applied with additional consideration of product format, palatability, species-specific safety, and the regulatory status of the intended use. Thus, method and carrier selection should be based on the minimum carrier complexity required to achieve the defined product function under practical manufacturing and regulatory constraints.

## 8. Conclusions

Encapsulation has evolved from a primarily protective formulation approach into an application-driven engineering strategy for designing functional food ingredients with improved stability, processability, sensory quality, and targeted delivery. Evidence from research publications and patents demonstrates that successful encapsulation depends on the rational integration of ingredient characteristics, carrier functionality, food matrix, processing conditions, storage environment, and intended release performance. Established processing methods, including spray drying, freeze drying, ionic gelation, and coating, remain important because of their scalability and manufacturing compatibility, while carrier systems such as emulsions, liposomes, and cyclodextrin inclusion complexes provide different functions in ingredient protection and delivery. Emerging carrier systems, including nano-enabled delivery systems and hybrid multilayer structures, together with fabrication methods such as electrohydrodynamic processing and microfluidics, provide greater control over carrier architecture and functionality, although broader industrial implementation still requires further advances in process scalability, economic feasibility, long-term stability, regulatory compliance, and commercial validation. Rather than seeking a universally superior method or carrier system, future developments should focus on application-oriented design in which encapsulation approaches are selected and optimized according to the specific requirements of the target food product and manufacturing process. Greater integration of food engineering, material science, ingredient functionality, and industrial processing will be essential for translating laboratory-scale innovations into commercially viable food applications. This review also highlights that specialized food systems, including pet food, should be considered as application-specific platforms requiring adaptation of general encapsulation principles rather than direct transfer of a carrier system or processing method. Future research should therefore emphasize matrix-specific validation, industrial-scale processing, shelf-life performance, sensory acceptance, and regulatory considerations to support the broader implementation of encapsulated functional ingredients across diverse food systems.

## Figures and Tables

**Table 1:** Classification and characteristics of encapsulation methods and carrier systems in food systems.

Method/Carrier System	Category	Typical Carrier Material	Main Function	Main Limitation
Spray drying	Fabrication method	Maltodextrin; gum arabic; modified starch; proteins	Powder formation; active protection	Thermal stress
Freeze drying	Drying method	Proteins; sugars; hydrocolloids	Preservation of heat-sensitive actives	High cost; low throughput
Coacervation	Fabrication method	Gelatin, chitosan, alginate, CMC	Encapsulation; controlled release	pH/ionic-strength sensitivity
Emulsion-based encapsulation	Formulation method	Lipids, proteins, surfactants	Dispersion of lipophilic actives	Coalescence; phase separation
Ionic gelation	Fabrication method	Alginate; pectin; chitosan	Mild encapsulation; controlled release	Limited mechanical strength
Extrusion encapsulation	Fabrication method	Alginate; starch; chitosan; carrageenan	High loading; structured delivery	Limited particle-size control
Fluidized-bed coating	Coating method	Lipids, polymers, proteins	Surface protection; post-process delivery	Equipment/coating control
Liposomes	Carrier system	Phospholipids	Hydrophilic/lipophilic delivery	Stability; oxidation; cost
Cyclodextrin inclusion complex	Carrier system	α-, β-, γ-cyclodextrins	Solubility improvement; flavor masking	Limited loading capacity
Nanoemulsion	Carrier system	Lipids, surfactants, biopolymers	Hydrophobic-active dispersion	Physical stability; processing energy
Nanoliposome	Carrier system	Phospholipids	Bioactive delivery	Stability; scalability
Solid lipid nanoparticles (SLN)	Carrier system	Triglycerides; fatty acids	Protection; controlled release	Limited loading capacity; polymorphic instability
Nanostructured lipid carriers (NLC)	Carrier system	Solid and liquid lipids	Improved loading; lipid delivery	Formulation complexity
Electrospraying/electrohydrodynamic processing	Fabrication method	Biopolymers, proteins, polysaccharides	Controlled particle formation	Low throughput; scale-up limitation
Microfluidics	Fabrication method	Polymers, lipids, proteins	Uniform particle formation	Cost; limited industrial scale
Hybrid/multilayer systems	Carrier architecture	Protein–polysaccharide; lipid–polymer	Multifunctional protection/release	Formulation complexity

**Table 4:** Representative research evidence on encapsulation and coating applications in pet food.

Product Format	Active/Intervention	Method/Carrier	Study Condition	Reported Outcome	Ref.
Dry kibble	Organic acid mixture containing HMTBa	Post-processing coating; canola oil and dry digest flavor carriers	Post-extrusion microbial contamination/storage	Reduced *Salmonella* and STEC; residual anti-Salmonella activity during storage; antifungal effect against *A. flavus* less consistent	[20]
Dry kibble	β-Hydroxy-β-methylbutyrate in free acid or calcium salt form	Post-processing coating with liquid HMBFA or powdered calcium HMB	Post-process *Salmonella* challenge	All HMB treatments eliminated detectable *Salmonella* within 14 days	[26]
Raw meat-based dog diet	Encapsulated citric and lactic acids	Encapsulated acidulants using protective carrier	1.0% acid treatment	Reduced *Salmonella* while preserving product quality better than dry-plated acidulants	[27]
Dry kibble	Menhaden fish oil	Post-processing oil coating	Temperature-dependent *Salmonella* challenge	Reduced *Salmonella* more effectively than canola oil, especially under elevated holding conditions	[25]
Dry kibble	Medium-chain fatty acids: caproic, caprylic, and capric acids	Post-processing fat coating	Post-extrusion *Salmonella* challenge	Individual MCFAs reduced *Salmonella* by >4.5 log within 15 h; some combinations reduced counts to nondetectable levels	[21]
Dried dog snacks	Canine-specific probiotics including *Lactobacillus* sp. Pom1 and *Limosilactobacillus fermentum* Pom5	Surface hydrogel coating; sodium alginate ± glycerol	28-day chilled storage and simulated GI conditions	Alginate–glycerol coating gave highest probiotic survival and improved simulated gastric/intestinal survival	[24]
Dog supplement powder	Canine-associated probiotic LAB strains	Extrusion microencapsulation; sodium alginate, goat milk, and β-glucan	Freeze drying, 2-month storage, and simulated gastric conditions	Encapsulation efficiency was 88.25–99.33%; improved survival after freeze drying and storage; gastric protection reported	[28]

## Data Availability

No new data were created or analyzed in this study. Data sharing is not applicable.

## Supplementary Materials

The following supporting information can be downloaded at: https://www.mdpi.com/article/10.3390/foods15183175/s1, Table S1. Complete patent inventory of established food encapsulation and coating methods and carrier systems. Table S2. Complete patent inventory of emerging food encapsulation methods and carrier systems. Table S3. Complete patent inventory of food encapsulation applications by active-compound group. Table S4. Patent-derived application summary for encapsulation and coating approaches relevant to pet food.
