# Supplementary material for: From Encapsulation to Food Delivery: Application-Driven Design of Functional Ingredients Using Encapsulation and Coating Approaches for Future Food Systems
Source: Foods. 2026 Sep 8;15(18):3175. doi: 10.3390/foods15183175 (PMC13605368; doi:10.3390/foods15183175)
Supplement: Supplementary file 1 [file foods-15-03175-s001.zip › foods-4538075-supplementary.pdf]

Supplementary Materials

Table S1. Complete patent inventory of established food encapsulation and coating methods and carrier systems.

NO.	Method and carrier system	Key process or design feature	Claimed food function	Main limitation	Relevance to specialized food applications	Ref.
**Spray drying**						
US20260096991	Spray-dried plant-protein hydrogel microcapsules; plant protein hydrogel shell or matrix	Plant protein is dissolved in a co-solvent system; pH is adjusted ≥0.5 unit below the isoelectric point; sol–gel transition and shear form hydrogel slurry; active is added before drying	Protection of thermally sensitive actives; aqueous and acidic formulation stability; possible intestinal release	Requires solvent, pH, and shear control; spray-drying stress may still affect highly sensitive actives; performance depends on active–protein compatibility	Relevant to supplement powders, liquid additives, coating premixes, wet-food inclusions, and probiotic or micronutrient delivery	[12]
US20260096575	Spray-dried enzymatically modified plant protein extract; plant protein carrier	Plant proteins are extracted under alkaline conditions; protein deamidase improves solubility; recovered protein suspension is spray dried	Improved protein solubility, dispersibility, emulsification, and foaming; compatible with dry ingredient production	Mainly improves carrier functionality rather than true active encapsulation; limited controlled-release role	Relevant to protein enrichment, functional powders, wet foods, high-protein products, and supplement blends	[13]
WO2026065618	Spray-dried chlorogenic-acid-rich coffee extract; no defined wall material	Green coffee extract is stabilized with vitamin C; caffeine is reduced by cation exchange resin; extract is spray dried at inlet 50–70 °C and outlet 50–60 °C	Stabilized antioxidant powder; reduced caffeine; improved flavor profile; scalable powder production	Not a true core–shell encapsulation system; spray drying mainly supports powder formation	Possible relevance to antioxidant premixes or functional additives, with limited evidence for controlled release	[10]
**Liposome encapsulation**						

NO.	Method and carrier system	Key process or design feature	Claimed food function	Main limitation	Relevance to specialized food applications	Ref.
US20260083675	Liposome encapsulation combined with freeze drying; phospholipid and chitosan system	Probiotic liposomes are formed with soybean phosphatidylcholine and modified phytosterol; modified chitosan is applied as a secondary coating; final powder is freeze dried	Improved probiotic survival during storage and simulated digestion; enhanced encapsulation efficiency; synbiotic powder format	Multi-step preparation; organic solvents and ultrasonic processing; freeze drying increases cost and processing time	Relevant to probiotic supplements, milk replacers, gastrointestinal-support powders, and freeze-dried functional products	[40]
RU0002858886	Bioactive peptide powder by enzymatic hydrolysis and drying; maltodextrin or whey protein carrier	Cattle plasma proteins are enzymatically hydrolyzed at 50–60 °C; peptides are fractionated; powder is produced by spray drying at 130 °C or freeze drying at −40 °C	Stable antioxidant peptide powder; improved handling and storage; suitable for dry or liquid formulations	Not a defined microcapsule system; animal blood plasma may raise sensory and regulatory concerns in some markets	Relevant to high-protein powders, recovery nutrition, functional liquid foods, and supplement formats	[41]
**Freeze drying**						
RU0002858875	Freeze-dried fungal biomass and culture fractions; fungal polysaccharide matrix	Cordyceps militaris is cultivated by submerged fermentation; biomass and culture liquid are separated, concentrated, and freeze dried to 6–10% moisture	Preservation of heat-sensitive fungal bioactives; water-soluble powder for food, feed, and supplement use	Energy-intensive process; no defined encapsulating shell; moisture-sensitive powder requires packaging control	Relevant to functional powders, feed premixes, and supplement formats when ingredient suitability is confirmed	[39]
**Fluidized-bed coating**						

NO.	Method and carrier system	Key process or design feature	Claimed food function	Main limitation	Relevance to specialized food applications	Ref.
WO2024246566	Fluidized-bed gastro-resistant pellet coating; polymeric barrier layers	Sodium bicarbonate pellets are coated with an insulating layer and a gastro-resistant outer layer; shellac or anionic methacrylate copolymer controls release	Reduced gastric release; improved intestinal delivery; lower coating load than some multilayer systems	Optimized for sodium bicarbonate and solid dosage forms; requires coating-thickness and pH-responsive polymer control	Relevant to solid supplements or functional particles requiring delayed intestinal release	[43]
US20260090562	Fluidized coating or curtain coating; flowable edible coating	A vertical coating curtain is formed through a dispensing slit ≤250 μm; products pass continuously through the curtain	Uniform coating thickness; controlled coating weight; continuous surface application	Surface coating rather than microencapsulation; requires precise pressure and flow control	Relevant to surface delivery of flavors, lipids, palatability compounds, or functional coatings in low-moisture foods	[46]
US20260090563	Multilayer hard-panning coating; enzymatically modified starch derivatives	Edible cores are coated through repeated syrup application and drying; coating syrup contains 0.5–7 wt% modified starch derivative	Titanium dioxide-free whitening; improved coating opacity, drying behavior, and dust control	Requires multiple coating and drying cycles; mainly a surface coating system	Possible relevance to coated snacks, confectionery-type foods, treats, and palatability-enhancing outer layers	[47]
US20260114475	Low-temperature fat-based surface coating for dry pet food; fat and palatability enhancers	Dry kibble receives a fat-containing first coating and liquid or powder palatability enhancers; coating is applied below about 37 °C	Improved palatability; late-stage aroma and flavor delivery; reduced heat exposure of palatants	Mainly surface delivery, not sealed encapsulation; coating uniformity depends on fat level and temperature	Relevant to post-processing finishing, aroma retention, and surface delivery in low-moisture foods, including dry pet foods	[95]
**Surface coating**						

NO.	Method and carrier system	Key process or design feature	Claimed food function	Main limitation	Relevance to specialized food applications	Ref.
US20260114489	Dry particle coating; hydrophobic stearate surface layer	Host powder particles are coated with hydrophobic stearate under high shear; guest particles are applied at about 1–10 wt%	Improved powder flowability; reduced caking; better moisture and oxygen resistance	Mainly surface protection; coating efficiency depends on particle size and mixing condition	Relevant to dry premixes, dusting powders, flavor powders, supplement powders, and surface-applied coatings	[44]
WO2026077956	Starch–fat edible surface coating; starch and plant oil system	Food cores are coated with starch, fat, and water; optional second coating contains ≥60 wt% gelatinized starch; coated product is heated at 120–300 °C	Texture control; fat retention in coating; reduced oil uptake and oxidation in heated foods	Structural coating rather than active encapsulation; heat process limits sensitive actives	Possible relevance to baked snacks, coated foods, texture-modified products, and lower-fat coated formats	[42]
EP4725323	Solid-fat shell encapsulation; fat-based shell for liquid fillings	Liquid core is enclosed in a solid fat shell; tempered cacao butter or related fats are melted, cooled, and solidified around the filling	Leakage reduction; stable liquid-filled product; texture and sensory control	Mainly designed for filled foods, not nutrient delivery; shell stability depends on fat crystallization and storage temperature	Possible relevance to filled foods, functional centers, or chewable formats when fat composition and storage stability are suitable	[45]
**Emulsion-based encapsulation**						
WO2021170619	Room-temperature emulsion coating; oil-in-water emulsion on low-moisture carrier	Flavor oil emulsion is mixed with an insoluble carrier at 20–30 °C; emulsion viscosity is >500 mPa·s at 25 °C; carrier water activity is <0.6	Improved volatile retention; reduced thermal flavor loss; high flavor loading	Limited to low-moisture carriers; physical stability depends on carrier water activity; flavor migration may occur	Relevant to flavor coatings, supplement powders, dry toppers, and surface flavoring in low-moisture foods	[51]
WO2026069317	Lipid-based micronized particle	Edible solids are dispersed in lipid or	Improved suspendability, flavor	Requires strict rheology control;	Relevant to lipid coatings, palatability enhancers, semi-	[50]

NO.	Method and carrier system	Key process or design feature	Claimed food function	Main limitation	Relevance to specialized food applications	Ref.
	suspension; oil-phase particulate dispersion	oil; particles are milled to <15 μm or <10 μm D50; final suspension contains ≥30 wt% solids; viscosity remains <10,000 cP	release, coating uniformity, and high solid loading in lipid systems	multi-stage milling increases equipment demand; oxidation risk during processing	moist products, nutrient suspensions, and supplement pastes	
US20260091023	Lipid matrix encapsulation in gel-chew system; lipid beads in hydrogel matrix	Active is dispersed in molten lipid matrix at about 70 °C; lipid beads are solidified and embedded in a gel matrix prepared at 40–70 °C	Protection of moisture-sensitive actives; taste masking potential; delivery of incompatible compounds in soft chews	Multi-component formulation; melting and cooling must be controlled; uniform bead distribution may be difficult	Relevant to chewable supplements, functional gel snacks, and semi-moist delivery formats	[48]
			**Inclusion complexes**			
US20260115248	Cyclodextrin inclusion complex; γ-cyclodextrin with botanical extract	Purified black ginger extract is mixed with γ-cyclodextrin in water; mixture is concentrated, freeze dried, and pulverized	Improved water and acid solubility; reduced bitterness and astringency; food-compatible powder format	Requires extract purification and composition control; mainly improves solubility and taste, not controlled release	Relevant to botanical powders, supplement formats, and wet-food applications requiring taste masking, with dose and target-use validation	[49]

Table S2. Complete patent inventory of emerging food encapsulation methods and carrier systems.

NO.	Method and carrier system	Key process or design feature	Claimed function	Main limitation	Relevance to specialized food applications	Ref.
WO2026089741	Oil-in-water flavor nanoemulsion; gum acacia, pectin, and weighting agents	Aqueous and oil phases are prepared separately; citrus oil and flavor compounds are homogenized by rotor–stator mixing and ultrasonication; droplet size is about 0.5–1.0 μm; processing is conducted at 0–6 °C under reduced oxygen	Citrus flavor and aroma delivery; reduced oxidation of oil-phase volatiles; improved shelf-stable flavor system	Requires high-shear and ultrasonic homogenization; oxygen control is needed; freezing may destabilize the emulsion; mainly designed for flavor delivery	Relevant to beverage, wet-food, coating, and flavor systems requiring volatile protection and aroma retention; product-format, palatability, and storage validation are needed.	[19]
US20260115146	protein-based nanocomplex; zein and food proteins	Water-soluble protein is dispersed in aqueous solvent; pH is adjusted to >11; zein and poorly soluble phytochemical are added; pH is adjusted to 5–8 for protein refolding; final particle size is <1 μm, preferably 40–200 nm	Improved water dispersibility of poorly soluble phytochemicals; physical protection; oxidative stability; dry-powder redispersion	Strong alkaline treatment may not suit all actives; protein–phytochemical compatibility may vary; allergen and labeling issues may apply	Relevant to supplement powders, wet-food inclusions, beverages, and functional powder blends requiring improved phytochemical dispersion	[18]
WO2026078444	Botanical nanoencapsulation; biodegradable polymers, liposomes, lipid nanoparticles, nanoemulsions, or cyclic dextrin systems	*Mitragyna speciosa* leaf is extracted using acidified water, alcohol, or acetone; extract is purified and standardized; nanosystem is formed	Improved solubility and delivery potential of lipophilic botanical alkaloids; standardized extract format; beverage or	Regulatory restrictions vary by region; safety and dose control are critical; multiple extraction, purification, and	Limited direct relevance to food applications where botanical safety, dose control, and regulatory status are uncertain; useful mainly as a model for botanical nano-delivery	[52]

NO.	Method and carrier system	Key process or design feature	Claimed function	Main limitation	Relevance to specialized food applications	Ref.
		by ultrasonication or high-pressure homogenization; solvent is removed by evaporation or lyophilization	supplement compatibility	nanoencapsulation steps are required		
US20260090963	Polymer-encapsulated pigment particles; hybrid micro-nano coating system	Xanthommatin or ommochrome pigments are stabilized in polymeric or inorganic particles; particles are produced at micro or nano scale; pigments are applied to edible substrates by spray coating, electrostatic coating, dipping, drum coating, pan coating, or fluidized-bed coating	Improved pigment stability and dispersion; pH-responsive and redox-responsive color behavior; flexible coating and incorporation formats	Primarily a pigment stabilization and coating system, not a nutrient-delivery platform; color depends on pH, oxidation state, and concentration; food and pet-food validation remain limited	Possible relevance to colored coatings, soft products, and wet-food systems requiring pigment stabilization and controlled appearance	[55]
US12599569	Polymeric-coated resveratrol nanoparticles; gum arabic shell and polyvinyl alcohol outer coating	Resveratrol is encapsulated with gum arabic and coated with polyvinyl alcohol; ratio is about 1:5:5 w/w/w; particles are prepared by nanoprecipitation and homogenization;	Core–shell nanoparticle formation; improved aqueous stability and delivery potential of poorly soluble resveratrol	Uses alcohol-based nanoprecipitation; polyvinyl alcohol may limit clean-label positioning; therapeutic or bioavailability claims need validation	Possible relevance to functional powders or supplement formats when resveratrol use is justified, dose-controlled, and suitable for the target product.	[53]

NO.	Method and carrier system	Key process or design feature	Claimed function	Main limitation	Relevance to specialized food applications	Ref.
		average size is about 195–210 nm				
WO2026075621	Liquid peptide condensate; shell-free microbial encapsulation based on liquid–liquid phase separation	*Lactobacillus*-derived REP6 peptide forms liquid condensates in aqueous solution; condensates aggregate and associate with probiotic or microbial cells; association is controlled by pH and environmental conditions	pH-responsive microbial protection; enhanced microbial aggregation and mucosal adhesion; probiotic viability under simulated gastrointestinal conditions	Early-stage platform; peptide engineering and production may be complex; food-scale validation and regulatory acceptance remain unclear	Potential relevance to probiotic supplements and specialized food formats, but product-matrix validation is still needed	[54]
WO2026072011	Electrospun nanofiber encapsulation; uniaxial or coaxial polymeric nanofibers	Active is incorporated into solutions, emulsions, or double emulsions containing 1–5 wt% active, 5–30 wt% polymer, and 65–94 wt% solvent; fibers are produced at 0.5–1.5 mL/h, 5–35 kV, and 5–20 cm collector distance	Sustained release; reduced burst release; adjustable multilayer nanofiber architecture; flexible incorporation of vitamins, antioxidants, oils, minerals, peptides, and plant extracts	Requires specialized equipment and parameter control; solvent and humidity affect fiber stability; mainly designed for transdermal delivery, not conventional oral food systems	Limited direct use in conventional food matrices; possible value in specialized supplement films or delivery formats requiring controlled release	[15]

Table S3. Complete patent inventory of food encapsulation applications by active-compound group.

Patent	Active group and formulation challenge	Method and carrier system	Key process or design feature	Claimed function in human food	Application-oriented interpretation	Ref.
**Lipid / omega-3**						
US20210037866	Lipophilic bioactives; poor emulsion stability, oxidation, phase separation, and low dispersibility	Emulsion-based encapsulation using physically modified flour as emulsifier and carrier; protein–starch matrix	Flour is milled for 2 min–50 h and heated at 40–300 °C for 2 min–100 h; oil-in-water emulsion is homogenized before dehydration	Improved emulsion stability, reduced creaming, better freeze–thaw stability, and improved dispersion of oleoresins and lipophilic nutrients	Useful for omega-3 powders, oil premixes, wet-food emulsions, milk-replacer-type products, and functional treats; product-matrix validation is still needed	[60]
CA3132477	Lipophilic compounds and edible oils; oil oxidation, poor dispersion, and unstable oil-in-water systems	Spray-dried microencapsulation using paramylon from *Euglena* as encapsulating polysaccharide	Oil is homogenized with paramylon and spray dried; encapsulation efficiency reported at ≥50%	Improved oil emulsification, viscosity, water binding, and redispersion after drying	Relevant to oil-containing powders, coating premixes, milk replacers, wet-food emulsions, and supplements when carrier suitability is confirmed	[80]
WO2024200171	Algal proteins and hydrophobic flavor compounds; weak creaminess, poor mouthfeel, and limited flavor delivery in plant-based foods	Algal protein coacervates and core–shell microparticles; polysaccharide shell materials	Coacervation and protein microparticle formation with optional cross-linking	Improved texture, mouthfeel, perceived creaminess, and flavor retention	Relevant to wet foods, gravy systems, soft products, and palatability systems, especially in plant-forward formulations	[81]
US20250302764	Algae-derived bioactives and micronutrients; poor solubility, oxidation, enzymatic degradation, and strong odor	Solid lipid nanoparticles; lipid matrix with surfactant-stabilized dispersion	Lipid matrix is dispersed and sonicated to form nanoparticles; surfactants stabilize particle uniformity	Improved stability against oxidation, heat, light, and digestion; sustained release and reduced sensory impact	Possible use in high-value supplements and lipid-based functional products; complexity and dose validation remain important limits	[16]
WO2017197453	Omega-3 polyunsaturated fatty acids; oxidation,	Spray drying; complex coacervation; multicore	Oil-in-water emulsion droplets are coated with protein and polysaccharide	Improved oxidative stability, reduced oil	Relevant to omega-3 fortification in dry, baked, wet, and coated	[56]

Patent	Active group and formulation challenge	Method and carrier system	Key process or design feature	Claimed function in human food	Application-oriented interpretation	Ref.
	rancidity, off-flavor, and instability of concentrated oils	coacervate microcapsules	wall materials before drying	exposure, and improved emulsion stability	food formats where oxidation control is needed	
US20200022394	Polyunsaturated fatty acid salts; fishy odor, poor dispersion, and low loading in milk-based products	Spray-dried fatty acid salt dispersion; sodium caseinate and whey protein as dispersing and surface-active matrix	Fatty acid salts are spray dried without a conventional carbohydrate wall; powder is designed for rapid dispersion	Rapid dispersion in milk and aqueous beverages; improved flowability and oxidative stability	Relevant to liquid supplements, milk replacers, and recovery beverages; palatability and lipid loading need product-specific testing	[82]
US20240065979	Polyunsaturated fatty acids and lipid-soluble bioactives; oxidation, rancidity, and vitamin loss during storage	Micelle encapsulation with controlled Maillard-reaction coating; de-oiled lecithin and reducing sugar systems	Vacuum-assisted Maillard processing under controlled reaction conditions	Improved oxidative stability, reduced rancidity, and stable coated micelles	Relevant to lipid-fortified foods, soft chews, milk-replacer-type products, and lipid supplements; heat exposure during processing remains a concern	[83]
			**Polyphenol / botanical**			
WO2021016520	Cannabinoids and phytochemical nutraceuticals; poor oral bioavailability and limited gastrointestinal absorption	Liposomal encapsulation; phospholipid bilayer with coconut-derived medium-chain triglyceride oil and caprylic acid	High-speed homogenization forms liposomes; oil-in-water liposomal system supports hydrophobic compound delivery	Encapsulation efficiency ≥60%; improved retention time and absorption compared with non-liposomal cannabinoid oil	Possible relevance to oral gel, liquid topper, or soft-chew supplement formats; cannabinoid use requires strict safety and regulatory validation	[57]
WO2022162565	Hydrophobic flavonoids; poor water solubility, sedimentation, instability, and low bioaccessibility	Spray-dried flavonoid dispersion; phosphate-assisted solubilization; protein-based stabilizers	Flavonoids are solubilized with phosphate, neutralized, and spray dried into amorphous powders	Improved aqueous solubility, reduced crystallinity, better beverage stability, and increased in vitro bioaccessibility	Relevant to powdered supplements, gravies, wet foods, and hydration products; palatability and dose validation are needed	[59]
WO2022013548	Plant bioactives and micronutrients; bitter	Multilayer encapsulation; core–	Fluidized-bed coating; spray drying; alginate bead	Reduced taste impact; improved heat	Relevant to bitter botanical extracts,	[58]

Patent	Active group and formulation challenge	Method and carrier system	Key process or design feature	Claimed function in human food	Application-oriented interpretation	Ref.
	taste, degradation, oxidation, poor release, and nutrient loss	shell particles using alginate, cellulose derivatives, and optional biopolymers	encapsulation; controlled intestinal-release design	protection up to 160 °C; delayed gastric release and improved intestinal delivery	micronutrients, and functional premixes; controlled-release claims need target-use digestion data	
US20200179477	Cacao flavonoids and catechins; chemical instability, bitterness, low oral bioavailability, and need for modified release	Hot melt extrusion; polymeric solid dispersion and modified-release matrix	Polymer matrix is processed by hot melt extrusion and controlled cooling; hydrophobic and cellulose-based polymers act as carriers	Improved thermal and moisture stability; taste masking; sustained release in simulated gastrointestinal conditions	Relevant to bitter polyphenols in soft chews, supplements, and extruded premixes; heat tolerance and sensory acceptance need validation	[77]
US20210378973	Creatine and amino acid supplements; poor aqueous stability, degradation, unpleasant sensory properties, and need for delayed release	Protein-based microencapsulation; co-extrusion and aqueous-core microcapsule formation	Hydrolyzed whey protein shell and phosphate cross-linking form protein microcapsules	Improved stability of creatine in aqueous storage; reduced conversion to creatinine; improved gastrointestinal release and beverage protection	Relevant to recovery supplements, liquid supports, and amino-acid delivery systems; application should consider formulation pH and acceptance	[84]
US20220110867	Mixed bioactives, including cannabinoids, fish oil, probiotics, vitamins, and flavor compounds; oxidation, bitterness, poor dispersion, and phase separation	Multilayer particle encapsulation; water-in-oil-in-water double emulsion; polymeric shell coating	High-shear homogenization and ultrasonication are used to form multilayer or emulsion-based particles	Improved emulsion stability, reduced oxidation and moisture exposure, taste masking, delayed release, and improved retention during storage	Relevant to wet foods, gravies, semi-moist products, and soft chews as a model for multifunctional delivery	[87]
WO2024256662	Water-soluble and oil-soluble bioactives; poor retention in cheese, aggregation, and	Kappa-casein nanocapsules and phospholipid-based nanoencapsulation	Lipid film hydration, homogenization, vortex mixing, and shell formation are used for hydrophilic or lipophilic compounds	Improved encapsulation and retention in cheese curd; improved protection of lactoferrin and lipophilic	Relevant to milk replacers, liquid supplements, and high-moisture foods; casein	[85]

Patent	Active group and formulation challenge	Method and carrier system	Key process or design feature	Claimed function in human food	Application-oriented interpretation	Ref.
	oxidation in fortified milk			compounds during storage and digestion	systems may help retain sensitive nutrients	
US20250134153	Lipids and bioactive nutrients; poor emulsion stability, uncontrolled oil release, and limited flavor retention in plant-based foods	Mycelium-based multilayer encapsulation; fungal matrix with alginate, chitin, protein, and hydrocolloids	Bioactives are incorporated into fungal pellets; drying and freeze drying improve solid loading; mechanical disruption supports emulsion formation	Improved oil retention during cooking; controlled fat and flavor release; improved mouthfeel and emulsion stability	Relevant to alternative-protein foods and omega-3 delivery; safety, palatability, and target-use status need validation	[86]
**Probiotic**						
EP4042881	Probiotic microorganisms; loss of viability during storage, moisture exposure, gastric acid, enzymes, and heat processing	Multilayer microencapsulation; water-blocking and hydrophobic outer layers	Extrusion spheronization, granulation, and fluidized-bed spray coating are used to form coated probiotic particles	Improved probiotic survival during room-temperature storage; reduced leakage; improved resistance to gastric acid and moisture	Relevant to probiotic coatings, freeze-dried diets, treats, and supplements; survival during severe processing should be validated	[78]
US20220273571	Probiotic and synbiotic compounds; low probiotic viability, oxidative degradation of algal oil, and moisture sensitivity	Biomass-based entrapment; protein–carbohydrate matrix from plant biomass	Non-fermented plant matrix is used as a protective barrier and prebiotic carrier	Higher probiotic viability; improved resistance to oxygen, temperature, moisture, and pH stress; improved oxidative stability of algal oil	Relevant to probiotic toppers, freeze-dried products, treats, and dry foods; plant-biomass palatability should be tested	[88]
US20230320991	Probiotics; low survival during freeze drying, processing, storage, acid, and bile exposure	Spontaneous alginate hydrogel encapsulation during cultivation	Sodium alginate and calcium carbonate generate alginate hydrogel in situ; corn starch acts as structural stabilizer	Improved freeze-drying viability, heat tolerance at 60 °C, simulated gastrointestinal survival, and long-term shelf stability	Relevant to probiotic powders, coated products, and functional treats; useful for thermally sensitive probiotic delivery in low-moisture products	[90]
US20250302757	Probiotics; heat sensitivity, moisture	Lipid microencapsulation;	Probiotics are dispersed in low-melting lipid	More than 80–90% probiotic viability	Relevant to coated products, probiotic	[89]

Patent	Active group and formulation challenge	Method and carrier system	Key process or design feature	Claimed function in human food	Application-oriented interpretation	Ref.
	sensitivity, acidic gastric conditions, and incompatibility with antimicrobial nutraceuticals	melt-spray-congeal lipid matrix	excipients; lipid matrix is formed with fatty alcohols, glycerides, waxes, and lecithin	retained after encapsulation; improved stability at 55–58 °C and under humidity; controlled release possible	premixes, soft chews, and freeze-dried toppers; post-processing application is more realistic than extrusion exposure	
**Flavor / aroma / essential oil**						
US20210186073	Flavor compounds and essential oils; volatility, oxidation, hydrolysis, flavor fading, and off-flavor development	Seed-based encapsulation and sustained-release delivery; intact edible seeds as carriers	Flavor is infused into porous edible seeds by mild thermal loading	Improved oxidative stability, prolonged flavor retention, reduced flavor loss during drying, and delayed release during chewing	Relevant to treats, dental sticks, dry toppers, and aroma-enhanced coatings; safety and sensory suitability of seed carriers should be checked	[79]
US20240180220	Flavor and bioactive compounds; volatile loss, heat instability, oxidation, and reduced flavor intensity	Protein-based hydrogel encapsulation; spray-dried hydrogel particles	Protein emulsion is thermally gelled to form hydrogel microcapsules; dry particles are produced by spray drying or related drying methods	Improved retention of flavor compounds; improved heat stability; enhanced flavor intensity and prolonged sensory perception	Relevant to dry foods, baked treats, snacks, and palatability systems; protein-wall sensory impact should be considered	[91]
WO2024137186	Bitterness modifiers and flavor agents; bitterness and astringency from plant proteins and high-intensity sweeteners	Cyclodextrin inclusion complex; beta-cyclodextrin as inclusion carrier	Flavor modifier is complexed with beta-cyclodextrin to reduce bitterness and improve mouthfeel	Reduced bitterness and astringency; improved smoothness, roundness, and palatability of plant-protein formulations	Relevant to supplements, plant-protein formulations, and functional additives requiring taste masking	[93]
US20230149878	Flavor oils and hydrophobic bioactives; volatility, poor aqueous stability, and limited plant-based delivery	Complex coacervation; plant protein–polysaccharide core–shell microcapsules	Hydrophobic compounds are encapsulated in plant-based coacervate shells; optional chitosan, carrageenan, alginate, or pectin supports shell formation	Formation of stable core–shell microcapsules; improved protection of volatile compounds; improved heat and shear resistance	Relevant to semi-moist products, powdered supplements, palatability enhancers, and plant-forward foods	

Patent	Active group and formulation challenge	Method and carrier system	Key process or design feature	Claimed function in human food	Application-oriented interpretation	Ref.
WO2025125286	Flavor and texture modifiers; poor foamability, weak creaminess, and unstable flavor compounds in plant-protein systems	Complex coacervation and protein–polysaccharide microparticles	Plant proteins and polysaccharides form coacervate shells; optional cross-linking strengthens capsule structure	Improved foam capacity, creaminess, flavor retention, and stability of hydrophobic flavor compounds	Relevant to milk replacers, liquid toppers, mousse-style treats, and texture-modified foods	[92]

Table S4. Patent-derived application summary for encapsulation and coating approaches relevant to pet food.

Product format	Main formulation or processing challenge	Encapsulation or coating approach	Claimed function	Application-oriented interpretation	Ref.
Dry kibble and low-moisture extruded products	Heat exposure, surface stickiness, uneven coating, oil migration, volatile loss, and post-processing delivery of sensitive ingredients	Hydrocolloid-stabilized glaze, fat-containing coating, low-temperature lipid multilayer coating, freeze-dried surface inclusion, vacuum-assisted lipid coating, and probiotic or vitamin microcapsules	Improved coating uniformity, reduced clumping, aroma retention, palatability, protected delivery of functional additives, and improved retention of probiotics or fat-soluble nutrients	Shows that low-moisture extruded foods often require post-processing coating or protected addition rather than direct exposure of sensitive ingredients to extrusion	[22,23,95-102]
Soft-extruded, baked, and coated dry products	Thermal degradation of functional ingredients, herbal odor, additive instability during baking, and need for surface or gastric delivery	Post-cooling oil coating, gastric-soluble polymer coating, low-temperature coating, and microencapsulated vitamins or botanical particles	Reduced thermal exposure, odor masking, improved palatability, protected additive delivery, and gastric disintegration after ingestion	Indicates that lower-temperature processing and targeted surface coating may help adapt encapsulation systems to dry or baked specialized foods	[100,114,115]
Wet foods and high-moisture matrices	Poor dispersion of lipophilic actives, pigment loss, flavor retention, sterilization stress, bitterness, and semi-solid texture stability	Calcium-crosslinked emulsion gel, cyclodextrin inclusion, whey-protein-based emulsion, and polymerized whey-protein systems	Improved dispersion, color retention, antioxidant capacity, gel texture, taste masking, and feeding acceptance after processing	Supports the use of emulsion gels and inclusion systems when high-moisture foods require dispersion, texture control, and sensory masking	[103,104]
Powdered pet palatability enhancer	Preservation of natural flavor and nutrients; microbial spoilage and oxidative deterioration of enzyme-treated fish-viscera material; storage stability	Compound-enzyme pretreatment of freshwater fish viscera followed by flavor-enhancer addition and low-temperature drying; base powder coated with a sodium-alginate/fish-glue	Improved palatability and flavor; protection from oxygen and moisture; improved storage stability and retention of the coated palatant	Represents a powder-based palatability system in which enzymatic flavor development is combined with a protective coating to improve acceptance and storage stability. The patent supports use as a pet-food palatant rather	[109]

Product format	Main formulation or processing challenge	Encapsulation or coating approach	Claimed function	Application-oriented interpretation	Ref.
		encapsulating matrix and freeze dried		than a probiotic or dose-controlled supplement system.	
Soft chews, semi-moist snacks, and structured supplement formats	Lipid oxidation, moisture migration, poor palatability, texture loss, probiotic survival, and controlled dissolution or digestion time	Linseed-oil microcapsules, high-internal-phase fish-oil emulsion, polysaccharide surface coating, dual-layer pectin and dietary-fiber coating, and freeze-dried probiotic microcapsules	Improved lipid stability, printability, freshness, chew texture, probiotic stability, storage stability, palatability, and controlled disintegration or digestion behavior	Shows how encapsulation can combine protection, texture control, and voluntary intake in semi-moist or chewable specialized foods	[107,108,111-113]
Flavor, aroma, and palatability systems	Volatile loss, off-odor, weak coating adhesion, unstable aroma release, and sensory instability during storage	Fat-based coating, palatability powder, oil-in-water emulsion, freeze-dried surface inclusion, hydrocolloid coating, and inclusion-based taste masking	Improved aroma retention, coating adhesion, flavor stability, odor control, and feeding acceptance	Provides a sensory-driven example in which release behavior and acceptance are as important as physical protection	[22,95,97,98,109]
